# Reduction of corticosteroid use in outpatient treatment of exacerbated COPD - Study protocol for a randomized, double-blind, non-inferiority study, (The RECUT-trial)

**DOI:** 10.1186/s13063-019-3856-8

**Published:** 2019-12-16

**Authors:** Pascal Urwyler, Maria Boesing, Kristin Abig, Marco Cattaneo, Thomas Dieterle, Andreas Zeller, Herbert Bachler, Stefan Markun, Oliver Senn, Christoph Merlo, Stefan Essig, Elke Ullmer, Jonas Rutishauser, Macé M Schuurmans, Joerg Daniel Leuppi

**Affiliations:** 1 University Department of Medicine, Cantonal Hospital Baselland, Rheinstrasse 26, CH - 4410 Liestal, Switzerland; 2 Faculty of Medicine, University of Basel, Klingelbergstrasse 61, CH - 4056 Basel, Switzerland; 3 Department of Clinical Research, University of Basel, Schanzenstrasse 55, CH - 4031 Basel, Switzerland; 4 Centre for Primary Health Care, University of Basel, Rheinstrasse 26, CH - 4410 Liestal, Switzerland; 5 Tyrolean Society of General Medicine, Innrain 71/2, A - 6020 Innsbruck, Austria; 6 Medical University of Innsbruck, Innrain 52, A - 6020 Innsbruck, Austria; 7 Institute of Primary Care, University and University Hospital of Zurich, Pestalozzistrasse 24, CH - 8091 Zurich, Switzerland; 8 Institute of Primary and Community Care, Schwanenplatz 7, CH - 6004 Luzern, Switzerland; 9 Centre for Lung Diseases Bern, Schaenzlistrasse 39, CH - 3013 Bern, Switzerland; 10 Department of Medicine, Cantonal Hospital Baden, Im Ergel 1, CH - 5404 Baden, Switzerland; 11 Department of Medicine, Cantonal Hospital Winterthur, Brauerstrasse 15, CH - 8401 Winterthur, Switzerland

**Keywords:** COPD, AECOPD, Exacerbation, Primary care, Corticosteroids

## Abstract

**Background:**

Chronic obstructive pulmonary disease (COPD) is a major public health issue affecting approximately 4% to 7% of the Swiss population. According to current inpatient guidelines, systemic corticosteroids are important in the treatment of acute COPD exacerbations and should be given for 5 to 7 days. Several studies suggest that corticosteroids accelerate the recovery of FEV1 (forced expiratory volume in 1 second), enhance oxygenation, decrease the duration of hospitalization, and improve clinical outcomes. However, the additional therapeutic benefit regarding FEV1 recovery appears to be most apparent in the first 3 to 5 days. No data are available on the optimum duration of corticosteroid treatment in primary-care patients with acute COPD exacerbations. Given that many COPD patients are treated as outpatients, there is an urgent need to improve the evidence base on COPD management in this setting. The aim of this study is to investigate whether a 3-day treatment with orally administered corticosteroids is non-inferior to a 5-day treatment in acute exacerbations of COPD in a primary-care setting.

**Methods/design:**

This study is a prospective double-blind randomized controlled trial conducted in a primary-care setting. It is anticipated that 470 patients with acutely exacerbated COPD will be recruited. Participants are randomized to receive systemic corticosteroid treatment of 40 mg prednisone daily for 5 days (conventional arm, *n* = 235) or for 3 days followed by 2 days of placebo (experimental arm, *n* = 235). Antibiotic treatment for 7 days is given to all patients with CRP ≥ 50 mg/l, those with a known diagnosis of bronchiectasis, or those presenting with Anthonisen type I exacerbation. Additional treatment after inclusion is left at the discretion of the treating general practitioner. Follow-up visits are performed on days 3 and 7, followed by telephone interviews on days 30, 90, and 180 after inclusion in the study. The primary endpoint is the time to next exacerbation during the 6-month follow-up period.

**Discussion:**

The study is designed to assess whether a 3-day course of corticosteroid treatment is not inferior to the conventional 5-day treatment course in outpatients with exacerbated COPD regarding time to next exacerbation. Depending on the results, this trial may lead to a reduction in the cumulative corticosteroid dose in COPD patients.

**Trial registration:**

ClinicalTrials.gov, NCT02386735. Registered on 12 March 2015.

## Background

In Switzerland, approximately 4% to 7% of the population suffer from chronic obstructive pulmonary disease (COPD), which is characterized by irreversible airflow obstruction and inflammation of the respiratory tract [1]. It is a progressive disease and its acute exacerbations are associated with increased morbidity and mortality. Thus, it is a major public health issue [2]. Data from a Swiss COPD cohort treated by general practitioners (GPs) demonstrated that approximately one in four COPD patients per year requires pharmacological treatment for an acute exacerbation of COPD (AECOPD) [3, 4]. Furthermore, a Spanish cross-sectional study found a median number of two exacerbations per patient per year in a population of 1001 COPD patients treated in general practice [5]. According to current guidelines, the inhalation of short-acting beta-adrenergic agonists and anticholinergic agents, as well as systemic glucocorticoids (GCs) is considered to be the standard therapy for AECOPD. The recommended daily treatment dose is 40 mg prednisone over 5 days [6, 7]. Several studies suggest that GCs accelerate the recovery of the forced expiratory volume in 1 s (FEV1), decrease the duration of hospitalization, reduce treatment failure, and improve clinical outcomes [8–14]. The additional therapeutic benefit regarding FEV1 recovery, however, seems to be most apparent during the first 3 to 5 days of GC treatment [8, 9].

The side effects of long-term GC treatment are well known, but even short-term treatment may cause adverse effects, such as secondary infections, hyperglycemia, or psychiatric symptoms [15]. Furthermore, repeated short-term applications of GCs result in high cumulative doses in the long term, which are associated with a higher vertebral fracture risk [16] and muscle weakness [17]. Whilst, there is strong evidence for the beneficial effects of GCs in the treatment of AECOPD, due to the potential serious adverse effects of GCs, coupled with a population base with frequent COPD exacerbations, a reduction in GC administration may be beneficial.

In our previous hospital-based study REDUCE, we found that a short 5-day treatment with systemic steroids was not inferior with regard to re-exacerbation compared to a conventional 14-day treatment in patients presenting to emergency departments with AECOPD [6]. These findings led to revisions of international guidelines [7]. However, even though many patients with AECOPD are treated as outpatients, no data are available about the minimal necessary duration of corticosteroid treatment in a primary-care setting. A shorter treatment duration may be advantageous in reducing long-term corticosteroid related side effects, as well as potentially being more cost-effective.

### Rationale

In this research project, we are focusing on optimizing the treatment of AECOPD in primary care, where the majority of patients are treated. The primary objective of this study is to investigate whether a 3-day treatment with orally administered systemic corticosteroids is non-inferior to a 5-day treatment for AECOPD in a primary-care setting. The primary endpoint is time to re-exacerbation. The study also aims to evaluate whether it is possible to minimize the cumulative dose of systemic GCs in patients suffering from AECOPD, without depriving them of the benefits of an optimal medication. A secondary objective is to evaluate differences between the two corticosteroid treatment durations regarding effectiveness and safety. Parameters to be evaluated as secondary endpoints are cumulative steroid dose, side effects and complications of GC treatment, change in FEV1, clinical course assessed through the COPD assessment test (CAT), need for hospitalization during the index exacerbation or during the follow-up, and death from any cause.

## Methods/design

### Study design and setting

The RECUT trial is a prospective randomized double-blind placebo-controlled non-inferiority trial in a primary-care setting. The coordinating study center is in Liestal, Switzerland, which is where the principal and co-principal investigators, study coordinators, and study physicians are based. The coordinating study center organizes all global activities in connection with the trial, is responsible for data management, and supervises endpoint adjudication. The trial steering committee is also based here. The study is conducted in collaboration with GPs in Northwestern and Central Switzerland, as well as in the Innsbruck area, Austria, who identify potential participants and perform study-related assessments. A list of participating GPs is available from the corresponding author on reasonable request.

Based on a sample size calculation, a total of 470 patients will be enrolled, with a 1:1 allocation ratio to the experimental and conventional arms. Participating GPs assess the eligibility of patients with AECOPD and perform any necessary diagnostic tests. Patients who fulfill the eligibility criteria and who are willing to participate receive 40 mg of oral prednisone per day for either 5 days (standard treatment group) or 3 days followed by 2 days of placebo (experimental group). Antibiotics (amoxicillin/clavulanic acid, 625 mg three times a day for 7 days) are administered to all patients with a serum C-reactive protein (CRP) level ≥ 50 mg/l at any of the study visits, and also to those with a known diagnosis of bronchiectasis or to those presenting with Anthonisen-type-I exacerbations [18]. Additional initial treatment and further treatments during the follow-up are determined and documented by the treating GP. Participants are assessed with respect to the primary and secondary endpoints after 3 and 7 days by their treating GPs. The coordinating study center contacts patients by phone for further evaluations on days 30, 90, and 180. If a patient cannot give sufficient information in the phone interview regarding the endpoints, then their GP is interviewed.

### Patient characteristics

The first patient was enrolled into the study in August 2015 and recruitment is expected to conclude by September 2021.

#### Inclusion criteria

To be included, patients must meet all of the inclusion criteria:

- Age ≥ 40 years
- Signed informed consent
- History of ≥10 pack-years of smoking (past or present smokers)
- Airway obstruction, defined as FEV1 / forced vital capacity (FVC) ratio ≤ 70%
- Current AECOPD by clinical criteria, defined by the presence of at least two of the following:
  - Change of baseline dyspnea
  - Change of cough
  - Change of sputum quantity or purulence

#### Exclusion criteria

Patients are excluded if they meet any of the exclusion criteria:

- Initial need for hospitalization
- Previous enrollment into the current study
- Asthma/COPD overlap syndrome with a predominant asthma component
- Diagnosis of tuberculosis
- Severe coexisting disease with life expectancy < 6 months
- Known severe immunosuppression or immunosuppression after solid organ or stem cell transplantation
- Inability to follow study procedures, e.g. due to language problems, psychological disorders, dementia, etc.
- Participation in another study involving an investigational drug
- Women who are pregnant or breastfeeding
- Premenopausal women with insufficient contraception and anamnestic risk for pregnancy

### Blinding and randomization

Identical blister packs with daily doses of 40 mg prednisone for either 5 days (standard treatment arm) or 3 days followed by 2 days of placebo (interventional arm) are packed in a 1:1 ratio in the hospital pharmacy of the University Hospital Basel, Switzerland, in an environment regulated by Good Manufacturing Practice. Each blister pack is labelled with a computer-generated random alphanumeric code. A concealed envelope marked with this alphanumeric code on the outside contains the group allocation and is kept safe at the study center until the final data are analyzed. Depending on the expected number of eligible patients, each recruiting GP receives a certain number of the pre-randomized blister packs and hands them out to participating patients. Trial participants, GPs, outcome assessors, and data analysts are blinded to group allocation. Unblinding is permissible if necessary for the urgent medical treatment of a participant.

### Study intervention and assessments

**Fig. 1:**
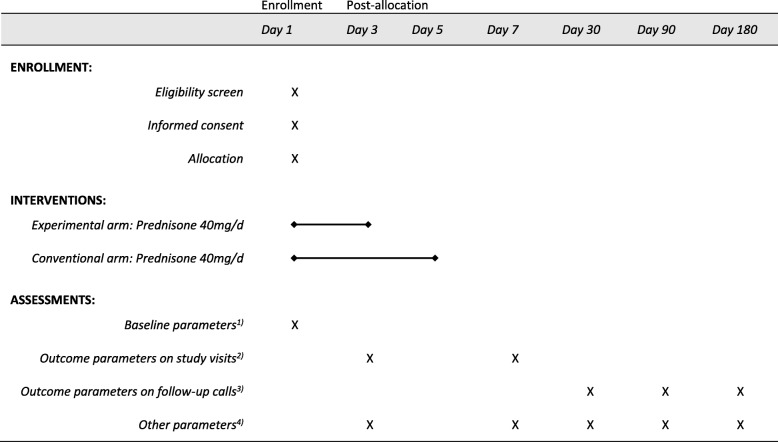
Summary of RECUT trial assessments performed at different time points. 1) Sex, age, weight, height, nationality, detailed medical history, CRP, plasma glucose, leucocyte cell count, respiratory rate, heart rate, blood pressure, pulse oximetry, body temperature, spirometry, mMRC, CAT, and quality and quantity of sputum and coughing. 2) All variables in 1) except demographic variables, but in addition treatment failure, hospitalization, mortality, change in medication, cumulative GC dose, clinically manifested side effects of GC or other medication. CAT only on day 7. 3) mMRC, CAT, quality and quantity of sputum and coughing, re-exacerbation, hospitalization, mortality, change in medication, cumulative GC dose, and clinically manifested side effects of GC or other medication. 4) Intervention (COPD self-management, smoking cessation), comments, and if lost to follow-up. CAT COPD assessment test, COPD chronic obstructive pulmonary disease, CRP C-reactive protein, GC glucocorticoid, mMRC Modified British Medical Research Council Dyspnea Scale

The schedule of enrollment, interventions, and assessments is presented in Fig. 1. On day 1 (inclusion visit), the treating GP informs any patients presenting with AECOPD about the trial, checks their eligibility criteria, gets written informed consent, and performs a general clinical assessment including vital signs, dyspnea assessment (using the Modified British Medical Research Council Dyspnea Scale, mMRC), and CAT. A blood sample for CRP, plasma glucose, and leucocyte cell count is taken and FEV1 and FEV1/FVC are assessed by spirometry. Participating patients receive 40 mg of oral prednisone per day for either 5 days (standard treatment group) or 3 days followed by 2 days of placebo (experimental group). Any additional newly started exacerbation medication besides the study medication will be documented. Treating doctors can re-evaluate and change the treatment at any stage of the trial if necessary (e.g., if the patient’s clinical condition worsens). The implementation of either trial arm will not require any alteration in usual care. Thus, all concomitant treatments or medications considered necessary by treating doctors, including any newly started exacerbation medication, are permitted and their use will be recorded in the case report form (CRF).

Follow-up visits will take place on day 3 (±1 day) and day 7 (±1 day) and each consists of a general clinical assessment, a blood sample, and an assessment of the clinical course regarding treatment failure and need for hospitalization. Furthermore, any changes in medication (including COPD baseline medication and exacerbation medication), cumulative GC dose, other interventions such as COPD self-management and smoking cessation, as well as clinically manifested side effects of GCs will be documented. During the second follow-up visit on day 7, a detailed medical history with regard to COPD is recorded and spirometry is performed. Participants are then followed up by phone on days 30, 90, and 180 after inclusion into the study (±7 days each). The phone interviews include dyspnea (mMRC) and CAT questionnaires, as well as questions regarding sputum, coughing, any changes in medication, and hospitalizations in the intervening time to assess re-exacerbation.

Participants may withdraw from the study at any time. Patients who prematurely withdraw from the study are nonetheless encouraged to attend the follow-up appointments. All data collected will be analyzed in an intention-to-treat analysis.

Since this study aims to evaluate a real-life situation in an outpatient setting as closely as possible and needs to be simple with regard to feasibility, patients’ adherence is not assessed. Patients are encouraged to return empty blisters to their GPs, which will be used to check whether they did take their medication according to the treatment plan. Poor compliance concerning the study medication is reported to the local study center and the coordinating center.

### Outcomes

The primary endpoint is time to next exacerbation during the 6-month follow-up period, which includes re-exacerbation during the index exacerbation (i.e. treatment failure). Exacerbation is defined as acute-onset worsening of the patient’s condition beyond day-to-day variations requiring interaction with a healthcare provider [19]. We chose time to next exacerbation as our primary endpoint to evaluate the effectiveness of the shorter steroid treatment. According to Leuppi et al. [6], Niewoehner et al. [9], and Aaron et al. [10], who investigated treatment failure rates, relapse rates, and time to relapse in AECOPD patients taking GCs, time to next exacerbation (which includes treatment failure) seems to be a valid measurement of effectiveness.

Secondary study outcomes are the cumulative GC dose, side effects and complications due to the GCs, change in FEV1, hospitalization rate during the index exacerbation and during the follow-up, clinical outcomes assessed by CAT and mMRC, as well as overall mortality. Cumulative GC dose and GC side effects are assessed to investigate the safety of short-term and standard steroid treatment. Furthermore, the change in FEV1, hospitalization rate during the index exacerbation and during the follow-up, as well as clinical outcomes and overall mortality are evaluated to compare the effectiveness of different durations of systemic corticosteroid treatment.

### Statistical analysis

It is hypothesized that the experimental treatment (3 days of corticosteroid treatment) is non-inferior to the conventional treatment (5 days of corticosteroid treatment) with regard to the primary endpoint. For this, a Cox proportional hazards regression model will be fitted to the data. Non-inferiority will be concluded if the two-sided 95% confidence interval of the hazard ratio between the experimental and the control arm lies entirely below the critical hazard ratio, which is defined as

$$ HR=\frac{\lambda_e}{\lambda_c}=\frac{\frac{-\log \left({\pi}_{et}\right)}{t}}{\frac{-\log \left({\pi}_{ct}\right)}{t}}=\frac{\log \left({\pi}_{et}\right)}{\log \left({\pi}_{ct}\right)}=\frac{\log \left({\pi}_{ct}-m\right)}{\log \left({\pi}_{ct}\right)} $$

where *t* is a fixed point of time, *λ*_*e*_ and *λ*_*c*_ are the hazard rates, *π*_*et*_ and *π*_*ct*_ are the proportions of event-free patients at time *t* in the experimental and conventional arms, respectively, and *m* is the non-inferiority margin, expressed as the additional proportion of patients having had an event in the experimental arm, assuming that the occurrence of events follows an exponential distribution [20]. This approach is partly based on the methodology described in a previous study undertaken by our research group [6, 21]. Following the recommendations of the Committee for Medicinal Products for Human Use, a two-sided 95% confidence interval is used to assess non-inferiority [22]. If there are missing data, GPs will be contacted by the study team with the aim of completing patients’ records, since data imputation is not planned. All statistical analyses will be performed on the per-protocol data set, complemented by a sensitivity analysis based on the intention-to-treat data set. Subgroup analyses or interim analyses are not planned.

### Sample size calculation

When estimating the sample size, we assumed an exacerbation rate of 30% to 40% following an exponential distribution and a 15% drop-out rate evenly distributed within the 6-month follow-up period, for both the interventional and the conventional arms. The non-inferiority margin was defined as a 15% increase of the exacerbation rate within 6 months. The significance level was chosen to be 5% and the power 80%. A simulation and a Cox proportional hazards regression model were used to determine hazard ratios and 95% confidence intervals for the simulated data sets, which led to a sample size of *N* = 466 (95% confidence interval 461–471) for an exacerbation rate of 30%, and *N* = 464 (95% confidence interval 459–469) for an exacerbation rate of 40%, respectively. Therefore, we aim to recruit *N* = 470 patients into the study. The sample size will be re-estimated after approximately half of the initially estimated number of patients have reached the 6-month follow-up. If necessary, the sample size will be increased. We will re-estimate the exacerbation rates in a blinded manner, based on the overall observed exacerbation rate as described by Friede et al. [23]. Since no hypothesis test will be performed, no *p*-value adjustment to control the type I error rate is needed. If it is anticipated that enrolment goals will not be met, a geographical expansion of the study will be considered.

### Safety and data security

Throughout the trial, all adverse events and serious adverse events will be recorded, fully investigated, and documented in source documents and CRFs. GPs are obliged to report serious adverse events within 24 h to the sponsor-investigator and the local project leader, who must report any deaths to the local ethics committee within 7 days. Adverse events and serious adverse events are followed up until resolution or stabilization. Important protocol modifications will be communicated to the relevant parties via email newsletters and personal phone calls.

All patient data are treated confidentially and are stored and analyzed in a coded way. Personal contact information, which is needed for follow-up phone calls, is stored separately and accessible only by the staff members making these phone calls. Information on data monitoring and auditing can be found in the SPIRIT checklist (Additional file 1).

## Discussion

The treatment of COPD, and especially the management of AECOPD, remains challenging in a primary healthcare setting. Practitioners aim to provide their patients with the most effective, yet safe and economical therapy, preferably with the fewest side effects. There is sufficient evidence that GCs have a positive effect on recovery from and clinical outcomes of AECOPD [10–14], with the current guidelines recommending a prednisone pulse of 40 mg daily for 5 days [6, 7]. However, the minimal effective duration of a GC pulse in AECOPD has not yet been determined. This is important since GCs may cause long-term side effects, and repeated short-term treatments have an impact on the cumulative dose. When AECOPD is treated in an outpatient setting, it can generally be assumed that it is less severe than in an in-hospital setting. Thus, a shorter GC treatment duration might be just as effective, but with fewer side effects. A reduction in the standard treatment duration could lead to significantly lower cumulative GC doses, especially in individuals with frequent exacerbations, and reduce the short- and long-term side effects. Furthermore, COPD-related healthcare cost could be reduced.

The high prevalence and mortality of COPD and its significant impact on quality of life implies that there is a need not only for prevention and new treatment options, but also for established treatment strategies to be optimized to reduce its overall burden. This strongly underlines the clinical relevance and importance of the RECUT trial. Furthermore, despite the availability of international guidelines, studies indicate that there is sub-optimal adherence to evidence-based COPD treatment strategies by GPs [3, 24]. Before advocating for a set of guidelines in primary care, the guidelines need to be verified within and optimized for this specific setting to increase their acceptability among practitioners and to ensure the best evidence-based treatment is given to patients. One of the key strengths of this project is its innovative design in a primary-care setting. The trial may also enhance awareness of the current guidelines and therefore, improve adherence to evidence-based treatment strategies among participating GPs. Further strengths of the study include its prospective, randomized, placebo-controlled, and double-blind design, as well as its relatively straightforward process. Even though a randomized approach was chosen, treating GPs retain control because, in accordance with the protocol, they can prescribe additional initial and follow-up treatments, which may enhance their willingness to collaborate.

### Trial status

The first patient was enrolled into the study in August 2015. The study is currently ongoing with active recruitment continuing under protocol version 5, dated 14 March 2019. Recruitment is anticipated to be complete by September 2021.

## Supplementary information

**Additional file 1.** SPIRIT 2013 checklist: Recommended items to address in a clinical trial protocol and related documents.

## Data Availability

Trial information can be found at ClinicalTrials.gov, NCT02386735. A completed SPIRIT checklist is available in Additional file 1. Data and materials that support this protocol, such as a detailed data management plan, CRFs, and informed consent form, are available from the authors on reasonable request.

## Abbreviations

AECOPD: Acute exacerbation of chronic obstructive pulmonary disease
CAT: COPD assessment test
COPD: Chronic obstructive pulmonary disease
CRF: Case report form
CRP: C-reactive protein
EKNZ: Ethics committee for the Region of Northwestern and Central Switzerland
FEV1: Forced expiratory volume in 1 s
FVC: Forced vital capacity
GC: Glucocorticoid
GP: General practitioner
mMRC: Modified British Medical Research Council Dyspnea Scale
